# ARCHITECT rHTLV-I/II immunoassay for blood screening and diagnosis

**DOI:** 10.1186/1742-4690-8-S1-A254

**Published:** 2011-06-06

**Authors:** Hans-Peter Kapprell, Michael Oer, Boris Heinrich, Carsten Buenning

**Affiliations:** 1 Abbott GmbH Co KG, Wiesbaden, Germany

## Background

Detection of HTLV infection continues to be important to control spread of human retroviruses and to protect safety of blood supply. The ARCHITECT rHTLV-I/II assay is a chemiluminescence-based immunoassay, which utilizes HTLV-I, and HTLV-II recombinant antigens and peptides in a double-antigen-sandwich assay format on the Abbott ARCHITECT automated platform. Precision, sensitivity and specificity of the ARCHITECT rHTLV-I/II assay is being presented.

## Methods

Assay performance was evaluated on confirmed HTLV-I/II positive specimens and specimens from German Blood Donors and hospitalized patients. External performance evaluation was conducted at two different sites in Portugal. Murex HTLV-I/II EIA was used as comparator assay and INNO-LIA or MP diagnostics HTLV Blot were used for confirmation.

## Results

The ARCHITECT rHTLV-I/II assay detected all HTLV-I and HTLV-II specimens (N=406) in accordance to the Murex assay. Analytical sensitivity was equivalent to Murex based on end-point-dilutions. Specificity on HTLV-I/II blood donors was 99.95% (N=5646; 95% CI 99.84-99.99%), SD to cut-off was 15.6. Specificity on a diagnostic population was 99.86% (N= 692; CI 95% 99.20-100%). Precision for samples (1-6 S/CO) was 3.98-4.31%. Presented data support selected cut-off multiplier-value of 0.25 for the ARCHITECT rHTLV-I/II assay. Results from the Receiver-Operating-Curve showed a 99.98% area under the curve which is indicative of high separation power between negative and positive populations.

## Conclusions

This study demonstrated excellent sensitivity and specificity of the ARCHITECT rHTLV-I/II assay. The assay is suitable for both blood screening and diagnosis of HTLV infections and therefore reduces risk of transfusion transmitted HTLV and improves safety of blood supply.

